# The hidden Markov model and its applications in bioinformatics analysis

**DOI:** 10.1016/j.gendis.2025.101729

**Published:** 2025-06-22

**Authors:** Yingnan Ma, Haiyan Chen, Jingxuan Kang, Xuying Guo, Chen Sun, Jing Xu, Junxian Tao, Siyu Wei, Yu Dong, Hongsheng Tian, Wenhua Lv, Zhe Jia, Shuo Bi, Zhenwei Shang, Chen Zhang, Hongchao Lv, Yongshuai Jiang, Mingming Zhang

**Affiliations:** a College of Bioinformatics Science and Technology, Harbin Medical University, Harbin, Heilongjiang 150086, China; b The Funome Project, Harbin, Heilongjiang 150086, China

**Keywords:** Copy number variation detection, CpG island prediction, Gene finding, Hidden Markov models, Sequence alignment, Transmembrane protein prediction

## Abstract

Big biological data contains a large amount of life science information, yet extracting meaningful insights from this data remains a complex challenge. The hidden Markov model (HMM), a statistical model widely utilized in machine learning, has proven effective in addressing various problems in bioinformatics. Despite its broad applicability, a more detailed and comprehensive discussion is needed regarding the specific ways in which HMMs are employed in this field. This review provides an overview of the HMM, including its fundamental concepts, the three canonical problems associated with it, and the relevant algorithms used for their resolution. The discussion emphasizes the model's significant applications in bioinformatics, particularly in areas such as transmembrane protein prediction, gene discovery, sequence alignment, CpG island detection, and copy number variation analysis. Finally, the strengths and limitations of the HMM are discussed, and its prospects in bioinformatics are predicted. HMMs can play a pivotal role in addressing complex biological problems and advancing our understanding of biological sequences and systems. This review can provide bioinformatics researchers with comprehensive information on HMM and guide their work.

## Introduction

Today, hidden Markov models (HMMs) are distinguished among the numerous statistical methods and algorithms employed in bioinformatics. HMMs are statistical frameworks designed to represent a Markov process with hidden, unobservable states. Owing to their capacity to capture dependencies between adjacent symbols, HMMs are inherently well-suited for sequence-related analyses and have been extensively utilized in bioinformatics applications since the 1980s.^1^

HMMs were initially utilized for protein structure prediction, where they demonstrated significant success in correctly identifying α-helices and β-barrel configurations in transmembrane proteins.^2^^,^^3^ Soon after, HMMs were widely adopted for genome annotation and powered gene prediction tools such as GENSCAN,^4^ which continue to exhibit strong performance today.^5^ Furthermore, HMMs were extensively applied to multiple sequence alignment and form the foundation of the Pfam database.^6^ With the completion of the Human Genome Project and the rapid advancement of high-throughput sequencing technologies, the availability of large-scale genomic datasets shifted the application of HMMs from traditional sequence modeling to the interpretation of complex genomic signals.^7^^,^^8^ During this period, HMMs found novel applications in Cytosine-guanine di-nucleotide (CpG) island prediction^9^^,^^10^ and copy number variation (CNV) detection,^11^^,^^12^ further reinforcing their significance in bioinformatics. In addition, HMMs are well suited for genetic mapping,^13^ phylogenetic analysis,^14^ and signal peptide prediction.^15^ Despite being such a powerful and widely used tool, HMMs still lack a clear and accessible introduction that matches their significance in the field.

In this paper, we systematically introduce HMMs, including their definitions, the three fundamental problems, and the corresponding algorithms. After outlining the core concepts of HMMs, we further examine their applications across five key areas of bioinformatics: transmembrane protein prediction, gene finding, multiple sequence alignment, CpG island prediction, and CNV detection, along with the commonly employed tools in each domain. Our objective is to offer readers a structured and comprehensive understanding of HMMs, thereby fostering a deeper appreciation of their underlying principles and diverse applications.

## Hidden Markov model and the relevant concepts

### Introduction of the hidden Markov model

**Figure 1:**
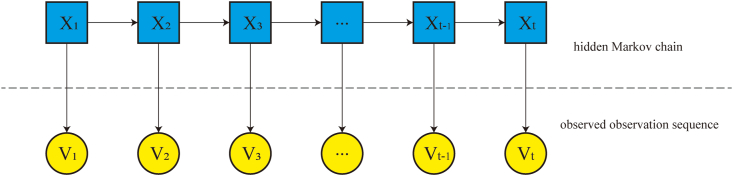
The two stochastic processes in the hidden Markov model: the generation of hidden states and their corresponding observations. The top panel shows the hidden state sequence, whereas the bottom panel represents the observation sequence.

HMMs, developed by Baum and associates in the 1960s, are statistical models that describe double-embedded stochastic processes, in which a hidden Markov chain controls the generation of observable data^16^, ^17^, ^18^ (Fig. 1). HMMs are widely used in modeling sequence data owing to their ability to describe complex relationships between hidden and observable variables.^19^ They are based on two key assumptions:

**Homogeneous Markov property**: The state at time *t* depends only on the state at time *t* – 1, and it is independent of any previous state or observation.

**Observation independence**: Observations depend only on the current state and are independent of any other state or observation.

For an in-depth discussion of the theoretical principles and algorithmic approaches of HMMs, readers are directed to Vidyasagar's monograph, *Hidden Markov Processes: Theory and Applications to Biology* (2014)*.* This work is an invaluable resource for researchers in systems theory and bioinformatics, providing a detailed examination of both the theoretical and algorithmic dimensions of HMMs.^20^

### Hidden Markov model parameters

i) **State space (*Q*)**: The set of all possible states, *Q* = {*q*_1_, *q*_2_ …, *q*_*N*_}, where *N* is the number of states.
ii) **State sequence (*X*)**: A sequence of states of length *T*, denoted as *X* = (*x*_1_, *x*_2_ …, *x*_*T*_).
iii) **Observation space (*V*)**: The set of all possible observable symbols, *V* = {*v*_1_, *v*_2_ …, *v*_*M*_}, where *M* is the number of possible symbols.
iv) **Observation sequence (*O*)**: A sequence of observable symbols corresponding to the state sequence, *O* = (*o*_1_, *o*_2_, …, *o*_*T*_).
v) **Initial state distribution (*π*)**: The probability distribution over the states at time *t* = 1, *π*_*i*_ = *P* (*x*_1_ = *q*_*i*_), *i* = 1, 2 …, *N.*
vi) **Transition probability matrix (*A*)**: The probabilities of transition between states, a_*ij*_ = P (*x*_*t*+1_ = *q*_*j*_ | *x*_*t*_ = *q*_*i*_), resulting in an *N* × *N* matrix.
vii) **Emission probability matrix (*B*)**: The probabilities of emitting observable symbols given a state, *b*_*j*_(*k*) = *P* (*o*_*t*_ = *v*_*k*_ | *x*_*t*_ = *q*_*j*_), forming an *N* × *M* matrix.

HMMs offer a robust framework for modeling sequences, characterized by the parameter set λ = (*A*, *B*, *π*). The hidden state sequence is determined by *π* and *A*, whereas the observable sequence is governed by *B*.^21^

### Three basic problems and corresponding algorithms

As a statistical model, the HMM offers robust theoretical support for sequence modeling. In practical applications, it can address the following three fundamental problems:

i) **Evaluation problem**

Given an HMM λ = (*A*, *B*, *π*) and an observation sequence *O* = {*o*_1_, *o*_2_, …, *o*_*T*_}, the likelihood *P* (*O* | λ) is calculated, which represents the probability of the observation sequence under the given model λ. This problem is crucial for model evaluation and can be efficiently solved via the ***forward algorithm*** or its complementary counterpart, the ***backward algorithm***.

#### Forward algorithm

The forward algorithm efficiently calculates *P* (*O* | λ) (starting from the beginning of the sequence) through dynamic programming. The method introduces an auxiliary variable *α*_*t*_ (*i*) = *P* (*o*_1_, *o*_2_, …, *o*_*t*_, *x*_*t*_ = *q*_*i*_ | λ), which represents the probability of observing a partial sequence of emissions *o*_1_, *o*_2_, …, *o*_*t*_ and a state *x*_*t*_ = *q*_*i*_ at time *t*. The algorithm's detailed equations are as follows:

i) Initialization:

$$\alpha_{1}\left(i\right)=\pi_{i}\cdot b_{i}\left(o_{1}\right),i=1,2,...,N$$

ii) Recursion:

$$\alpha_{t+1}\left(j\right)=\left(\sum_{i=1}^{N}\alpha_{t}\left(i\right)\cdot a_{ij}\right)\cdot b_{j}\left(o_{t+1}\right),i=1,2,...,N;\;t=1,2,...,T-1$$

iii) Termination:

$$P\left(O|\lambda \right)=\sum_{i=1}^{N}\alpha_{T}\left(i\right)$$

#### Backward algorithm

The backward algorithm computes *P* (*O* | λ) by considering paths starting from the end of the sequence. The algorithm's detailed equations are as follows:

i) Initialization:

$$\beta_{T}\left(i\right)=1,i=1,2,...,N$$

ii) Recursion:

$$\beta_{t}\left(i\right)=\sum_{j=1}^{N}a_{ij}\cdot b_{j}\left(o_{t+1}\right)\cdot \beta_{t+1}\left(j\right),t=T-1,T-2,...,1$$

iii) Termination:

$$P\left(O|\lambda \right)=\sum_{i=1}^{N}\pi_{i}\cdot b_{i}\left(o_{1}\right)\cdot \beta_{1}\left(i\right)$$

ii) **Decoding problem**

Given λ and *O*, the most likely hidden state sequence *X* = {*x*_1_, *x*_2_, …, *x*_*T*_} is determined. This corresponds to finding the maximum a posteriori (MAP) estimate of the state sequence, which is typically solved using the ***Viterbi algorithm***.

#### Viterbi algorithm

The Viterbi algorithm finds the most likely state sequence *X*^∗^.^22^^,^^23^ This algorithm employs two auxiliary variables, *δ* and *Ψ*. *δ*_*t*_ (*i*) represents the probability of the most probable partial path reaching state *i* at time *t*. *Ψ*_*t*_ (*i*) records the preceding state of *i* at the end of the locally optimal path. *Ψ*_*t*_ (*i*) enables the reconstruction of the state sequence that yields the highest partial probability δ_*t*__+1_ (*i*) at time *t* + 1. The algorithm is composed of the following four main steps:

i) Initialization:

$$\delta_{1}\left(i\right)=\pi_{i}\cdot b_{i}\left(o_{1}\right),i=1,2,...,N$$

$$\psi_{1}\left(i\right)=0,i=1,2,...,N$$

ii) Recursion:

$$\delta_{t}\left(i\right)=\max_{1\leq j\leq N}\left(\delta_{t-1}\left(j\right)\cdot a_{ji}\right)\cdot b_{j}\left(o_{t}\right),i=1,2,...,N$$

$$\psi_{t}\left(i\right)=\arg\max_{1\leq j\leq N}\left(\delta_{t-1}\left(j\right)\cdot a_{ji}\right),i=1,2,...,N$$

iii) Termination:

$$P^{*}=\max_{1\leq i\leq N}\delta_{T}\left(i\right)$$

$$x_{T}^{*}=\arg\max_{1\leq i\leq N}\delta_{T}\left(i\right)$$

iv) Backtracking:

$$x_{t}^{*}=\psi_{t+1}\left(x_{t+1}^{*}\right),t=T-1,T-2,...,1$$

The states in the optimal path are obtained by backtracking the antecedent states according to the variable *Ψ*_*t*_ (*i*). Finally, we can obtain the optimal path *X*∗ = ($x_{1}^{*}$, ${\;x}_{2}^{*}$, …, ${\;x}_{T}^{*}$).

iii) **Learning problem**

The objective is to estimate the model, specifically the two parameters: the transition probability and the emission probability. Furthermore, it is assumed that the number of states, N, in the underlying HMM is known. The model parameters must be optimized to maximize *P* (*λ* | *O*).

#### Supervised learning algorithm

It is assumed that the training data contain both observation and state sequences. In this case, the parameters of the HMM are usually estimated based on frequency.

If state *i* is at time *t*, state *j* is at time *t*+1, and the frequency of transitions between states in the sample is *A*_*ij*_. The estimation of the state transition probability is estimated as follows:

$${\overset{\wedge }{a}}_{ij}=\frac{A_{ij}}{\sum_{j=1}^{N}A_{ij}},i=1,2,...,N;\;j=1,2,...,N$$

Estimation of the observation probability *b*_*j*_(*k*): If the frequency of state *j* emitting observation *k* in the sample is *B*_*j*_(*k*), then the probability of state *j* emitting observation *k* is as follows:

$${\overset{\wedge }{b}}_{j}\left(k\right)=\frac{B_{j}\left(k\right)}{\sum_{k=1}^{M}B_{j}\left(k\right)},j=1,2,...,N;\;k=1,2,...,M$$

The initial state probability *π*_*i*_ is estimated as the relative frequency of state q_*i*_ occurring at the initial position across the S samples.

#### Baum-Welch algorithm for unsupervised learning

When the training data include only observation sequences without corresponding state information, algorithms such as the Baum-Welch method, a specialized expectation-maximization algorithm, are employed.^24^, ^25^, ^26^ This algorithm iteratively refines parameter estimates to maximize the likelihood of the observed data. It alternates between:

i) **Expectation step (E-step)**: Compute the expected sufficient statistics for the hidden states using current parameters.
ii) **Maximization step (M-step)**: Update the parameters to maximize the likelihood.

An illustrative example using boxes and balls is provided in the supplementary materials to explain the HMM problems and algorithms in detail.

### The application and tools of HMM in bioinformatics

The HMM is a widely utilized modeling approach for linear problems, such as time series data and biological sequences. Initially applied in the field of speech recognition,^19^ it has proven particularly valuable for modeling biological sequences.^1^ Proteins and DNA, as essential biological macromolecules, are fundamentally represented by sequences. Typically, distinct substructures in a biological sequence correspond to specific functions, with different functional regions often exhibiting unique statistical characteristics. This is the statistical foundation underlying the success of HMMs and has proven to be very effective in analyzing biological sequences.^1^ We elaborate on how the three fundamental problems of HMMs are applied in bioinformatics from five application perspectives, and introduce representative tools commonly used in each. Details of the tools mentioned in the review are shown in the supplementary table.

### Transmembrane protein prediction

Transmembrane proteins are a class of proteins that span the phospholipid bilayer of cellular membranes and play critical roles in various biological processes.^27^ They function as channels for the transport of ions across membranes and serve as targets for numerous drug molecules, including those involved in nerve signaling, hormonal regulation, and receptor activity.^28^ The secondary structure of the majority of transmembrane proteins is alpha-helical, with beta-barrel structures representing a notable exception. These beta-barrel transmembrane proteins are currently found exclusively in gram-negative bacteria, mitochondria, and chloroplasts.^29^

To understand the specific functions of transmembrane proteins, we need to understand their topology, that is, the orientation of the transmembrane protein relative to the membrane and the number and specific positions of the transmembrane segment.^30^ However, predicting the structure of transmembrane proteins using chemical or physical methods remains challenging.^31^^,^^32^ Currently, known transmembrane protein structures represent only a small fraction of entries in the Protein Data Bank.^33^ Consequently, a variety of statistical and bioinformatics approaches for transmembrane protein prediction have been developed over the past decades.^34^ Among these, methods based on HMMs have demonstrated outstanding performance.^35^, ^36^, ^37^, ^38^, ^39^

**Figure 2:**
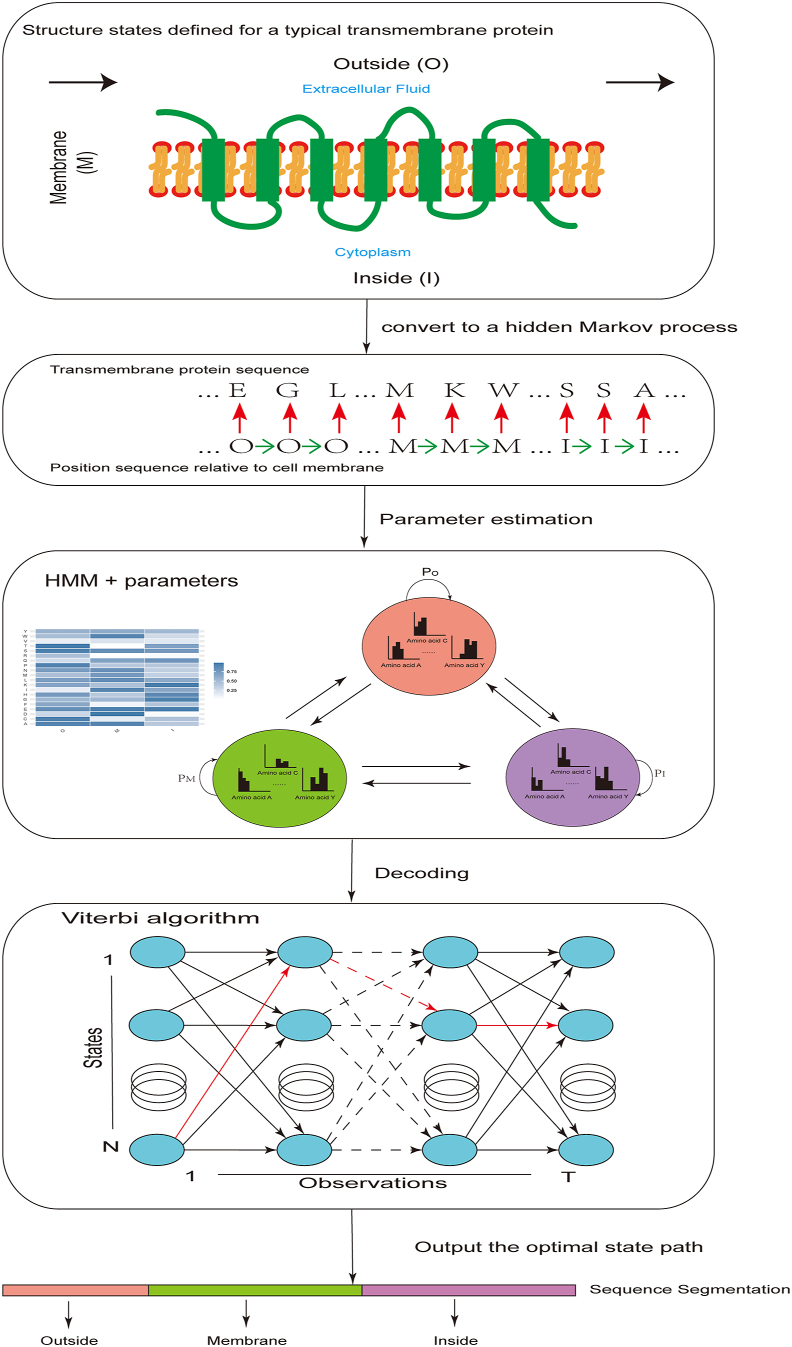
The decoding problem and Viterbi algorithm in transmembrane prediction. The amino acid sequence of the transmembrane protein and its corresponding positions on the cell membrane are transformed into a hidden Markov process. After evaluating the parameters, the Viterbi algorithm is used to identify the optimal state sequence.

HMMs were initially applied to the prediction of transmembrane proteins. This is a standard decoding problem that can be solved by the Viterbi algorithm in HMMs (Fig. 2). First, the structural states of a transmembrane protein need to be defined. According to its position relative to the cell membrane, a transmembrane protein can be simply divided into three states: Outside (O), Membrane (M), and Inside (I). In this way, it can be transformed into a hidden Markov process, namely, the hidden transmembrane protein position sequence and the visible amino acid observation sequence. Unlike other parts outside the membrane, the transmembrane segments of transmembrane proteins are hydrophobic. Therefore, amino acid residues with stronger hydrophobicity are more preferred to occur in the transmembrane segments. This provides a statistical basis for the recognition of transmembrane proteins. After evaluating the parameters of the HMM, the Viterbi algorithm can be used to identify the optimal state path of a new transmembrane protein.

HMMTOP is a powerful transmembrane protein prediction server that performs well in multiple comparisons with similar tools.^40^^,^^41^ Owing to its powerful properties, HMMTOP is one of the most used prediction tools in transmembrane protein-related studies. For example, Shao et al used HMMTOP2.0 to predict the transmembrane helical regions in the atomic models of the small capsid proteins P16, P17, and P18.^42^ Jiang et al used HMMTOP to predict the protein motifs of Cowpea mild mottle virus TGBp2 and reported that TGBp2 has two transmembrane domains near the N-terminus and C-terminus.^2^

HMM-TM (http://bioinformatics.biol.uoa.gr/HMM-TM) is a method for predicting transmembrane alpha-helical proteins that allows the integration of experimentally validated prior topological information specific to the sequences under analysis.^35^ Compared with HMMTOP, HMM-TM introduces enhancements to the prediction algorithm by incorporating precise positional information for segments of the predicted sequence, while maintaining the probabilistic framework essential for HMM decoding. Additionally, HMM-TM supports TMRPres2D, the Transmembrane Protein Re-Presentation in 2 Dimensions tool, which facilitates the automated generation of standardized, high-resolution two-dimensional graphical representations of alpha-helical transmembrane proteins.^43^

HMMpTM (http://bioinformatics.biol.uoa.gr/HMMpTM) is a transmembrane protein topology prediction tool based on HMM that combines post-translational modification and topology prediction of alpha-helical transmembrane proteins.^44^ Phosphorylation and glycosylation are the most common post-translational modifications in eukaryotes^45^ and occur in a specific manner in cells. In transmembrane proteins, glycosylation sites are usually located in the extramembrane space, whereas phosphorylation sites are located in the cytoplasmic region. Therefore, glycosylation and phosphorylation sites provide valuable information for predicting the orientation of transmembrane proteins relative to their membrane.^46^ Although HMMpTM was originally developed to predict the topological structure of transmembrane proteins, its capacity to identify phosphorylation and glycosylation sites is also noteworthy. Sara Savage et al classified HMMpTM as a predictive tool for phosphorylation sites and kinase substrates.^47^ Triantaphyllopoulos et al employed HMMpTM not only to predict transmembrane topology but also to identify potential phosphorylation and glycosylation sites in solute carrier family 11 A1 (SLC11A1).^48^

Currently, due to the absence of transmembrane helix predictors, signal peptides are frequently misidentified as transmembrane helices.^3^^,^^49^^,^^50^ This misprediction occurs because both transmembrane and signal peptide regions are characterized by the hydrophobicity of their residues, which is highly similar in both regions.^51^ Phobius (https://phobius.sbc.su.se/) is a combined predictor of transmembrane helices and signal peptides that is based on the HMM, which can overcome this limitation. In a recent study, Tirincsi et al used Phobius to predict the topological structure of a protein. They reported that the targeting receptor hSnd2 contains four transmembrane helices, with both the N-terminus and C-terminus in the cytosol.^52^

Beta-barrel proteins are more difficult to predict topologically than are alpha-helices. Although these methods have not been the focus of computational methods, they play important roles in bacteria, chloroplasts, and mitochondria.^53^ PRED-TMBB (http://bioinformatics.biol.uoa.gr/PRED-TMBB) is the first freely accessible HMM-based tool for predicting the topology of beta-barrel outer membrane proteins.^54^^,^^55^ In addition, Hayat et al^56^ and Tsaousis^57^ developed other HMM-based methods aimed at predicting beta-barrel transmembrane proteins.

### Gene finding

With the successful completion of the Human Genome Project^58^ and advances in sequencing technology, many measured but unannotated DNA sequences are generated daily. Efficient and accurate computational techniques are essential for annotating DNA sequences.^59^ Gene identification, a key challenge in genome annotation, involves detecting coding regions or genes within the sequenced DNA. After this problem is solved, further specific functional annotation of the genome can be performed.^60^

**Figure 3:**
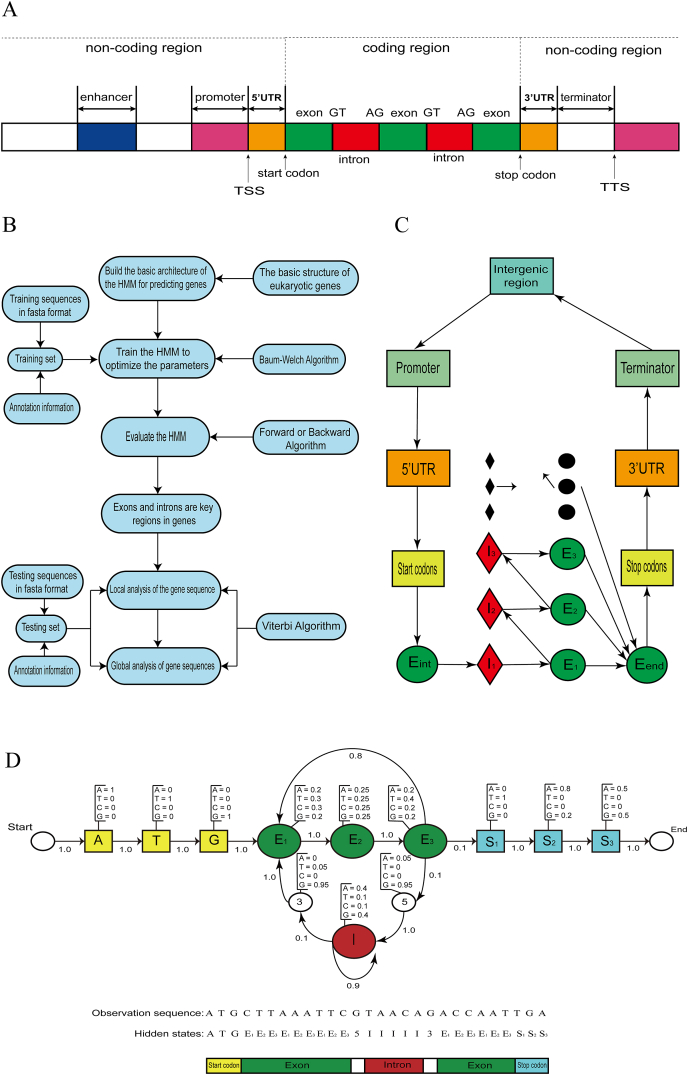
An example of a hidden Markov model (HMM). **(A)** Schematic diagram of eukaryotic gene structure. **(B)** The overall process of using the HMM to predict eukaryotic genes. **(C)** HMM architecture for predicting eukaryotic genes. **(D)** The submodel of the coding region.

Given the observed sequence of a protein-coding gene, we employed an HMM to predict the positions of exons, introns, and other critical functional regions and loci. This is also a decoding problem within the HMM framework, as the locations of these functional elements are not directly observable from the sequence. As shown in Figure 3A, eukaryotic genes are composed of coding and noncoding regions. Coding regions mainly contain exons and introns. Noncoding regions contain specific sequences and sites that play important roles in gene expression, such as enhancers, promoters, terminators, transcription start sites, and transcription termination sites. After outlining the structure of eukaryotic genes, HMM is employed for their prediction, with the detailed process depicted in Figure 3B. In Figure 3C, each rectangle, diamond, or circle represents a functional unit (state) of a gene or genomic region, including the intergenic region, promoter, terminator, 5′ untranslated region, 3′ untranslated region, start codon, stop codon, E_init_ initial exon, E_k_ (k = 1, 2, …) internal exon, E_term_ terminal exon, and I_k_ (k = 1, 2, …) introns.

We developed a specialized HMM submodel for the coding region (Fig. 3D), in which each square and circle denotes a hidden state, with the accompanying labels indicating their respective biological annotations. Each state can emit one of the four bases, A, C, T, or G, and its emission probability is indicated above it. The three yellow hidden states correspond to the start codon, which is ATG in eukaryotes; hence, they are labeled A, T, and G. The green hidden states represent exons, while the blue hidden states denote stop codons. Since mRNA is translated by codons comprising three bases, each codon encodes a specific amino acid. In the exon region, we further illustrate that the codon unit and the hidden states representing exons in the model form a cyclic structure. The red hidden state corresponds to an intron, characterized by a self-loop transition. The states labeled 5 and 3 represent the 5′ and 3′ splice sites, respectively. The start and end states do not emit any bases, but they are incorporated to complete the model structure. The hidden state sequence and state path are displayed below in Figure 3D.

GENSCAN (http://argonaute.mit.edu/GENSCAN.html) is an HMM-based program for the genetic prediction of the position and exon-intron structure of genes in genomic sequences from a variety of organisms.^4^^,^^61^ Additionally, it takes into account the significant variations in gene density and structure across different GC compositional regions of the human genome. GenScan employs a generalized hidden Markov model (GHMM)^62^ (where the state represents an arbitrary sub-model of the output variable length sequence) to create distinct sub-models tailored to the unique characteristics of each gene region. Each state can generate observations of a specific length based on a given probability distribution, greatly enhancing the accuracy and reliability of the predictions. As one of the most widely used gene-prediction tools, its results are highly precise.^63^^,^^64^ GENESCAN has been used to predict the gene structure of *Populus euphratica*^5^ and *Xingguo gray* goose genes.^65^

AUGUSTUS (http://bioinf.uni-greifswald.de/augustus/) predicts genes in eukaryotic genomic sequences based on a GHMM.^60^^,^^66^^,^^67^ It features a flexible mechanism for integrating external information, such as EST alignments and protein alignments, which can significantly enhance the accuracy of prediction results.^68^ It is powerful and has collaborated with many genome researchers.^69^^,^^70^ For example, Srivastava et al used AUGUSTUS to annotate protein-coding genes in *Amphimedon queenslandica*, helping explain the complexity of animal evolution.^71^

GeneMark (http://opal.biology.gatech.edu/GeneMark/) is a suite of gene prediction programs designed for the analysis of prokaryotic, eukaryotic, and viral genomic sequences.^72^ It enables accurate and efficient identification of genes within genomic DNA. GeneMark has been predominantly utilized for annotating prokaryotic genomes and played a pivotal role in the annotation of the first fully sequenced bacterial genome, *Haemophilus influenzae*, as well as the archaeal genome of *Methanococcus jannaschii*.

HMMGene (https://services.healthtech.dtu.dk/services/HMMgene-1.1/) is a program for the prediction of genes in anonymous DNA via HMM^1^ and is mainly used for vertebrate and *Caenorhabditis elegans* gene prediction.^73^ Krogh et al used the HMMGene gene finder to annotate the 3 MB Adh region of *Drosophila melanogaster* and reported that database matching significantly improved the performance of the gene finder.^74^

### Sequence alignment

Sequence alignment forms the foundation of sequence analysis and is one of the most fundamental and crucial techniques in bioinformatics. The goal of sequence alignment is to identify the maximum number of matching residues between two or more sequences using mathematical models or algorithms. The results of this comparison reflect the similarity between the sequences and their biological characteristics. Evolutionary theory serves as the theoretical basis for sequence alignment. Numerous biological observations demonstrate that different nucleic acid and protein sequences may have originated from a common sequence and evolved independently through genetic variation of residues. As a result, determining whether an organism's sequence belongs to a specific family is a common analysis in multiple sequence alignment.^75^ Sequence alignment can also identify conserved sequence fragments associated with structure and can be used for protein functional domain identification,^76^ secondary structure prediction,^77^ and phylogenetic analysis.^78^

**Figure 4:**
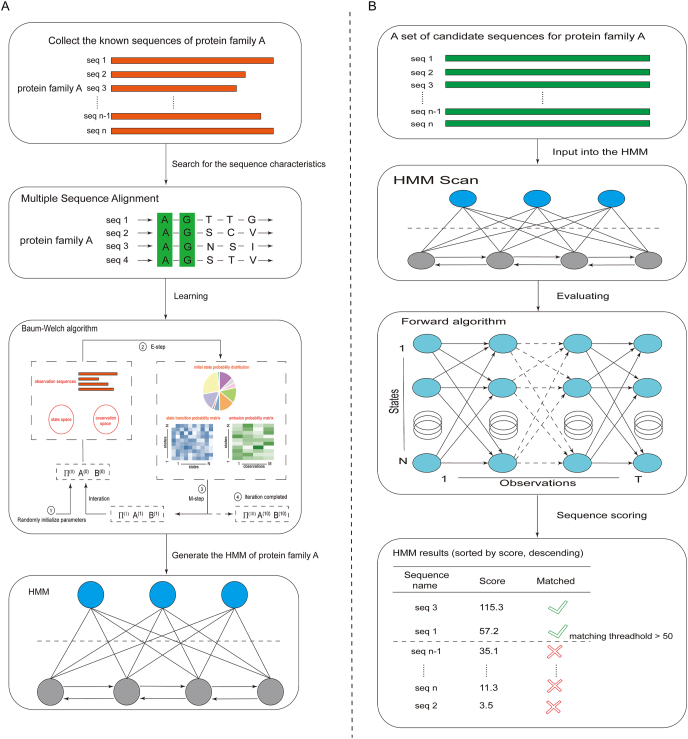
Applications of the learning and evaluation problems in protein family identification. **(A)** The learning problem and the Baum-Welch algorithm are used in constructing the hidden Markov model (HMM) of protein family A. After discovering the statistical characteristics of protein family A through multiple sequence alignment, the parameters were obtained with the help of the Baum-Welch algorithm to construct the HMM of protein family A. **(B)** The evaluation problem and forward algorithm are used in the identification of protein family A. A set of sequences is input into the HMM of protein family A, and each sequence is scored to determine whether it belongs to protein family A.

HMMs have an outstanding performance in the recognition of protein families. This exactly corresponds to the learning problem of HMMs, and it can be perfectly solved using the Baum-Welch algorithm (Fig. 4A). After collecting the known sequences of protein family A, through multiple sequence alignment, the conserved positions of the sequences belonging to the same protein family can be identified, and the statistical characteristics of the protein family can be constructed, which provides a basis for the subsequent construction of HMMs. The model parameters are estimated using the Baum-Welch algorithm, which is a form of the expectation-maximization algorithm. This algorithm first needs to initialize the parameters of the model, and then uses the method of maximizing expectations to continuously iterate the parameters of the model until the model stabilizes. Through the above operations, an HMM exclusive to protein family A can be obtained, which has a strong recognition ability for sequences belonging to protein family A.

After the HMM construction of protein family A is completed, in the face of a new set of unrecognized sequences, it is an evaluation problem to determine which sequences belong to protein family A. These sequences are input into the constructed HMM, and each sequence is scored through the forward algorithm. After setting a threshold, it is possible to determine which sequences belong to protein family A and which do not (Fig. 4B).

The HMMER web server (http://www.ebi.ac.uk/Tools/hmmer) is a collaborative project between the HMMER algorithm developers, led by Sean Eddy of Harvard University, and the HMMER Web Services team led by Rob Finn of EMBL-EBI. HMMER performs sequence homology searches in sequence databases and alignments using profile-hidden Markov models. Commonly used in conjunction with databases such as Pfam, HMMER facilitates sequence queries similar to those conducted with BLAST. The tool offers four search algorithms—phmmer, hmmscan, hmmsearch, and jackhmmer—each with distinct query and target database configurations. These algorithms allow users to define two types of thresholds: significance and reporting cut-offs. By inputting a sequence in FASTA format or uploading a file, users can select the target databases for the search. The output includes visual representations of sequence matches, annotated features, and the distribution of significant hits. HMMER supports searches across a wide range of target databases and integrates cross-referencing with resources hosted by EMBL-EBI. Additionally, it provides a comprehensive application programming interface and a dedicated category viewer for enhanced user interaction.^6^^,^^79^^,^^80^

Pfam (http://pfam.xfam.org/) is a database of protein family collections, each of which is represented by multiple sequence alignments and HMMs. A protein molecule comprises multiple structurally specific and functionally distinct regions, which serve as the fundamental units of protein functional domains. The function of a protein is determined by the combination of its domains, and proteins sharing the same domain typically belong to the same family. In this database, protein families are classified into families, clans, and proteomes. A clan refers to a group of families that share similar three-dimensional structures or common motifs. Proteomes provide comprehensive information on all protein families within a given species. Pfam consists of two primary components. The first part, Pfam-A, contains curated families, and each family has a related profile HMM that can be used for sequence alignment and database search. The protein family in each Pfam-A consists of four elements: i) annotation, ii) seed alignment, iii) profile HMM, and iv) complete alignment. To make Pfam more comprehensive, sequences not included in Pfam-A were automatically clustered in the second part, Pfam-B, via the Domainer algorithm. The Pfam database, version 37.1, contains 23,794 families and 751 clans.^81^, ^82^, ^83^

### CpG island detection

CpG sites are regions in the DNA sequence where guanine follows cytosine in the linear arrangement of nucleotides from the 5′ to 3′ direction. In mammals, CpG exists in two forms: one is dispersed throughout the DNA sequence, while the other is highly concentrated, referred to as CpG islands.^10^ In 1987, Gardiner-Garden and Frommer first described and defined CpG islands in detail. CpG islands have three main characteristics: i) they are more than 200 bp in length; ii) the GC content is more than 50%; and iii) the ratio of actual CpG content to expectations (ObsCpG/ExpCpG) is greater than 0.6.^84^ In 2002, Takai and Jones revised the definition as follows: the length should be more than 500 bp, the GC content should not be less than 55%, and the actual CpG content-to-expected ratio should be more than 0.65.^85^

CpGs have many important biological functions. It is a target of DNA methylation and is the main object of epigenetic research. In vertebrates, most CpG dinucleotides are dispersed, and the remaining CpGs tend to cluster in CpG island regions.^10^ CpG islands, which are frequently located near gene promoters, participate in the regulation of gene expression and are associated with cancer.^86^ In recent years, several HMM-based CpG island prediction methods have emerged.

CpG island prediction methods can be categorized into four main types: window-based, density-based, distance/length-based, and HMM-based approaches.^87^ Durbin et al proposed a specific methodology for applying HMMs to CpG island prediction.^1^ The HMM framework addresses two central problems in CpG island prediction: determining whether a short genomic sequence originates from a CpG island, and identifying CpG islands within long genomic sequences. For the first problem, two separate HMMs, model^+^ and model^–^, are constructed using sequences with known CpG and non-CpG islands, respectively. The combined model consists of four hidden states: A^+^, C^+^, T^+^, and G^+^. To accommodate sequence length modeling, the start and end states were incorporated into the HMM structure. The target regions of model^+^ were marked as CpG islands, and those of model^−^ were marked as non-CpG islands. In the model^+^, A^+^, C^+^, T^+^, and D^+^ emit corresponding bases with a probability of 1, whereas the start and end states do not emit any symbols. The details of the model^−^ are the same as those of model^+^. Finally, we calculate the score of a given sequence via the following formula:

$$Score\left(X\right)=\log\frac{P\left(X|CpG\;island\right)}{P\left(X|non\_CpG\;island\right)}=\sum_{i=1}^{L}\log\frac{a_{x_{i-1}x_{i}}^{+}}{a_{x_{i-1}x_{i}}^{-}}$$

Where L is the length of the given sequence, $a_{x_{i-1}x_{i}}^{+}$ is the transition probability of model^+^, and $a_{x_{i-1}x_{i}}^{-}$ is the transition probability of model^−^. The larger the score is, the more likely the sequence is to be a CpG island.

To address the second problem, it is necessary to integrate model^+^ and model^–^ to create a new model. This new model consists of eight states, with each state emitting a corresponding symbol. These eight states can transition between each other. After the training set was selected, the Baum-Welch algorithm was used to estimate the model parameters, and the Viterbi algorithm was applied to solve the optimal path.

The UCSC Genome Browser (https://genome.ucsc.edu/) is the browser most used to download CpG islands.^10^ The CpG island data were based on the definitions proposed by Gardiner-Garden and Frommer. An appropriate algorithm was designed to search for CpG island regions that met the criteria to produce the list. The Table Browser tool was used to download CpG island data in bulk from the Genome Browser database. The data included information such as the specific position on the chromosome, length, number of CpG dinucleotides, GC content, and the O/E ratio. The most prevalent form of DNA methylation involves the addition of a methyl group to the 5-position of cytosine within the 5′-C-phosphate-G-3' (CpG) dinucleotide, a process catalyzed by DNA methyltransferases. Methylated CpGs constitute about 70 %–80 % of all CpG sites in the human genome.^88^ In contrast, CpG islands located in the promoter regions of many highly expressed genes tend to remain unmethylated.^84^ The methylation status of CpG sites in promoter regions is closely associated with gene expression levels. Aberrant DNA methylation can lead to cell differentiation and is implicated in various pathological conditions, including cancer, mental disorders, and developmental abnormalities.^89^ DNA methylation primarily regulates gene expression through neighboring differentially methylated sites, which collectively form differentially methylated regions (DMRs). Currently, the two principal techniques used to assess genome-wide methylation status are methylation arrays and bisulfite sequencing.^90^ Many tools have been developed to detect DMRs based on the data detected by these two methods. Herein, we introduce several HMM-based tools for DMR detection.

DMRMark is a free R package on the Comprehensive R Archive Network (CRAN) that can detect DMRs from methylation array data based on a non-homogeneous hidden Markov model (NHMM). Chen et al developed a method based on NHMM to detect DMRs from bisulfite paired sequencing data and provided an R package called BSDMR. In both the case and control groups, the BDRMR model performs well in predicting DMRs in paired WGBS data from patients with colon cancer.^91^

Mao et al developed a new algorithm based on HMM to detect different regions of DNA methylation in MBDCap-seq data.^92^ ImaneSaif et al employed an HMM-based algorithm to predict DNA methylation in the promoter regions of tumor suppressor genes, aiming to provide early diagnosis for patients at risk of developing cancer.^93^

### Copy number variation detection

CNV is a type of structural variation in the genome characterized by segments greater than 1 kb in length.^94^ These variations result from genomic rearrangements, such as non-allelic homologous recombination, leading to duplications or deletions in the genomic structure, which correspond to gains or losses in copy number.^95^ As a significant form of genetic variation, CNVs are strongly associated with various diseases that have substantial impacts on human health, including neurological disorders, metabolic diseases, and cancer.^94^ Early detection of CNVs enables the identification of large-scale DNA sequence alterations in the genome, providing a critical foundation for the diagnosis and treatment of these conditions.

Next-generation sequencing offers a source of data for the detection of CNVs. Microarray technology can also be employed in detecting CNVs.^96^ CNV detection uses signal intensity data observed by microarrays to determine the hidden copy number of each locus in the genome. The three HMM-based methods commonly used for CNV detection utilizing log R ratio (LRR) and B allele frequency (BAF) data from microarrays are QuantiSNP,^97^ PennCNV,^98^ and GenoCN.^99^ These tools, similar to sequence-based approaches, employ HMMs to classify copy number alterations into three fundamental states: deletion, normal, and gain. By integrating emission probabilities derived from both LRR and BAF signals, these methods enable the accurate identification and characterization of CNVs across the genome. Several tools for detecting CNVs are described below.

ExomeDepth is an R package available through CRAN.^12^^,^^100^ It accepts input files in BAM and BED formats and incorporates GC content correction along with a beta-binomial model to mitigate noise in CNV detection. There are three principal approaches for detecting CNVs from short-read sequencing data: split reads, paired-end reads, and read depth, with the read depth method proving particularly effective for exome data. ExomeDepth applies a robust beta-binomial model to read depth data and constructs an optimized reference exome set. Each exon is classified into one of three states: deletion, normal, or duplication. An HMM is then employed to detect CNVs across multiple exons, with each state transition corresponding to a specific exon within the human genome. The Viterbi algorithm was used to call CNVs in the genome. Blanco-Verea et al used ExomeDepth to detect CNVs in genes from patients with familial heart disease and reported three true CNVs in five individuals, namely, myosin heavy chain 11 (*MYH11*), fibrillin 1 (*FBN1*), and *PDMI7*.^11^

XHMM is a suite of statistical and computational tools based on HMMs for detecting CNVs from exome sequencing data.^101^^,^^102^ Given the non-contiguous distribution of exons across the genome, depth of coverage serves as the primary source of information for CNV detection. However, noise and systematic biases complicate the interpretation of coverage data. XHMM addresses this challenge by employing PCA to reduce the influence of noise. The tool requires a C++ compiler and takes as input a reference genome in FASTA format, its corresponding BWA index file, and a list of exome targets in the “interval-list” format used by GATK. With a powerful HMM framework, XHMM can automatically call the copy number robustly and genotype CNVs across all samples. Lobon et al used XHMM to examine somatic CNVs in Parkinson's disease and reported that these mutations may affect genes that play a role in synaptic and neuronal processes.^103^

ExomeCopy (http://www.bioconductor.org/packages/2.12/bioc/html/exomeCopy.html) is an R package for detecting copy number variants via HMM. ExomeCopy employs a negative binomial model to adjust for factors such as read depth, GC content, and window width, while utilizing an HMM framework to detect CNVs. It accepts BED and BAM files as input. This method has demonstrated reliability across various exome enrichment platforms and in detecting a wide range of CNV types and sizes.^104^ Compared with standardized and state-of-the-art segmentation methods, ExomeCopy exhibits greater sensitivity, particularly in identifying overlapping minority exon duplications and heterozygous deletions in exome sequencing data. The tool formulates CNV detection as an optimization problem of the likelihood function over a limited set of parameters, thereby eliminating the need for arbitrary thresholding or preprocessing steps that could potentially impact downstream analyses. Overall, ExomeCopy is an excellent tool for detecting CNVs. Ravindran et al used it to detect CNVs in whole-exon sequencing data from Indian prostate cancer patients and revealed a new drug target, DNA polymerase theta (POLQ).^105^

## Discussion

The HMM is a statistical model developed from the Markov chain and was first used in speech recognition.^19^ HMM was subsequently introduced into bioinformatics because of its incredible potential for biological sequence analysis.^1^ The HMM is underpinned by a robust mathematical foundation and offers a powerful framework for modeling and analyzing biological sequences. It effectively captures the inherent randomness of biological variables while simultaneously simulating their structural characteristics. With its strong capacity to model internal dependencies and stochastic signals, the HMM has proven to be a highly effective tool for bioinformatics modeling and prediction. It has been successfully applied to a wide range of biological challenges, including transmembrane protein prediction, gene discovery, sequence alignment, CpG island prediction, and CNV detection. In recent years, HMMs have also been extended to tackle more complex problems in bioinformatics, such as modeling metabolic networks^106^ and integrating multi-omics data.^107^ Currently, the HMM is one of the most widely used methods in bioinformatics, and this review introduces the concept of the HMM and its application in bioinformatics.

HMMs can generally solve biological problems after a corresponding specific HMM architecture is designed. However, capturing higher-level information is challenging owing to the linear nature of the HMM. For instance, in protein structure prediction, two residues that are spatially proximate in the folded conformation may be widely separated in the linear amino acid sequence. Due to its inherent linear architecture, the HMM is unable to capture long-range dependencies, making it insufficient for accurately predicting spatial relationships between distant residues in folded proteins. Moreover, HMMs require substantial amounts of data to effectively estimate model parameters and mitigate the risk of overfitting.

Although the HMM has been extensively utilized in bioinformatics, there remains considerable potential for enhancing its performance and computational efficiency. As the field of bioinformatics continues to advance rapidly, emerging research areas and novel problems are continually introduced, necessitating the development of more sophisticated and adaptable modeling approaches. The HMM should evolve and adapt to current advancements and emerging challenges and be combined with other models and algorithms, such as Bayesian theory,^108^ artificial neural networks,^109^^,^^110^ and support vector machines.^111^ HMM will likely play a more important role in bioinformatics applications with the continuous development of science and technology in the future.

## CRediT authorship contribution statement

**Yingnan Ma:** Writing – review & editing, Writing – original draft. **Haiyan Chen:** Writing – review & editing, Supervision, Resources. **Jingxuan Kang:** Writing – review & editing, Resources. **Xuying Guo:** Writing – review & editing, Resources. **Chen Sun:** Data curation. **Jing Xu:** Formal analysis. **Junxian Tao:** Formal analysis. **Siyu Wei:** Methodology. **Yu Dong:** Methodology. **Hongsheng Tian:** Supervision. **Wenhua Lv:** Supervision. **Zhe Jia:** Validation. **Shuo Bi:** Validation. **Zhenwei Shang:** Methodology, Investigation. **Chen Zhang:** Visualization, Software. **Hongchao Lv:** Validation, Supervision. **Yongshuai Jiang:** Validation, Supervision, Funding acquisition. **Mingming Zhang:** Supervision, Funding acquisition, Formal analysis.

## Funding

This work was supported by the National Natural Science Foundation of China (No. 31970651, 92046018) and the Mathematical Tianyuan Fund of the National Natural Science Foundation of China (No. 12026414).

## Conflict of interests

The authors declared no competing interests.
