# Supplementary material for: The hidden Markov model and its applications in bioinformatics analysis
Source: Genes Dis. 2025 Jun 22;13(1):101729. doi: 10.1016/j.gendis.2025.101729 (PMC12505677; doi:10.1016/j.gendis.2025.101729)
Supplement: Multimedia component 1 [file mmc1.docx]

**Supplementary Material**

**Contents**

**[Graphical Explanation of HMM Algorithms 1](#_Toc24687)**

*[The Forward Algorithm](#_Toc11004)* [3](#_Toc11004)

*[The Backward Algorithm](#_Toc24103)* [5](#_Toc24103)

**[Illustrating HMM Through a Three-State Toy Example 6](#_Toc8863)**

*[Solving the Evaluation Problem with Forward Algorithm](#_Toc14336)* [9](#_Toc14336)

*[Optimal State Sequence with Viterbi Algorithm](#_Toc6512)* [10](#_Toc6512)

**[Implementing HMM Algorithms to the Toy Example in R 12](#_Toc29335)**

**[Supplementary introduction of HMM related tools 15](#_Toc2092)**

*[Transmembrane protein prediction](#_Toc22528)* [15](#_Toc22528)

*[Gene finding](#_Toc7097)* [19](#_Toc7097)

*[Sequence alignment](#_Toc18470)* [23](#_Toc18470)

*[CpG island prediction](#_Toc26757)* [24](#_Toc26757)

*[Copy number variation detection](#_Toc12581)* [25](#_Toc12581)

**[References 26](#_Toc18947)**

#

# Graphical Explanation of HMM Algorithms

HMM has a strong statistical theoretical basis. It is mainly used to deal with the following three problems which are also called the three basic problems. In the one-order HMM, a large number of different value states at each time point arise when moving from one time point to the next. Takes advantage of the power of the trellis diagram, the states of different variables can be arranged in an interlaced trellis diagram according to the position of the state sequence in the one-order HMM. The nodes of the trellis diagram correspond to a certain state at a certain position, and each node is connected to at least one node of the previous and the next variable at least one node. According to the conditional independence, each node can store computational information about all state sequences associated with it. The forward, backward and the Viterbi algorithms are based on the trellis diagram shown in **Figure S1.**


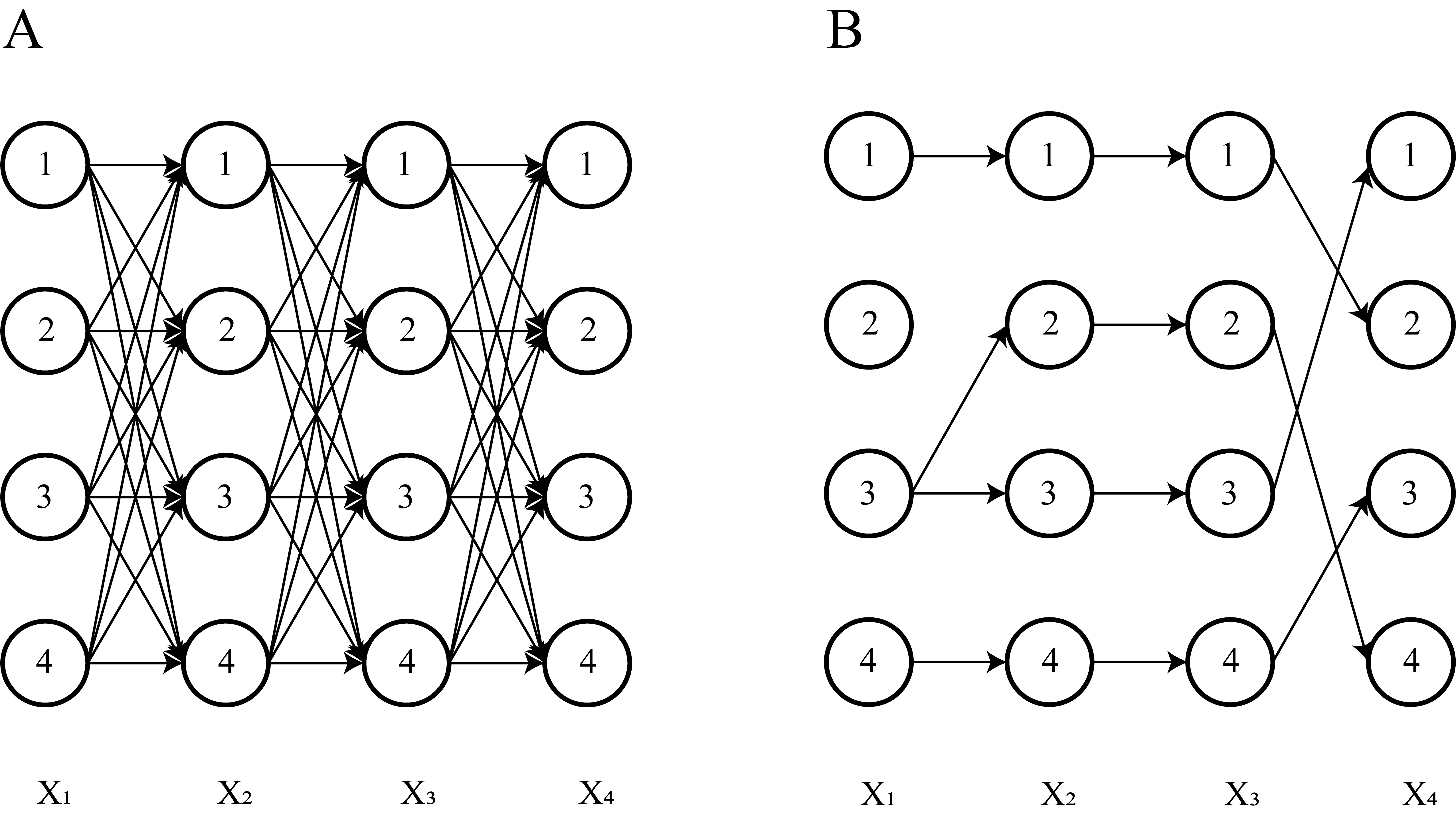


**Figure S1**. The trellis diagrams for the forward-backward algorithm and the Viterbi algorithm. (A) Trellis diagram of forward and backward algorithm. (B) Trellis diagram of Viterbi algorithm.

Both the forward and backward algorithms solve the evaluation problem of HMM. They include three steps: initialization, recursion, and termination. Each node in the trellis diagram has two variables to help store the forward probability *α_t_* (*i*) in **Figure S2 (A)** and the backward probability *β_t_* (*i*) in **Figure S2 (B)**. The *t* denotes the time point and the *i* denotes the state when it is located at *t*. In the trellis diagram, *α_t_* (*i*) and *β_t_* (*i*) denote the sum of the probabilities of all paths from the initial position to *i* and from *i* to the final position, respectively.


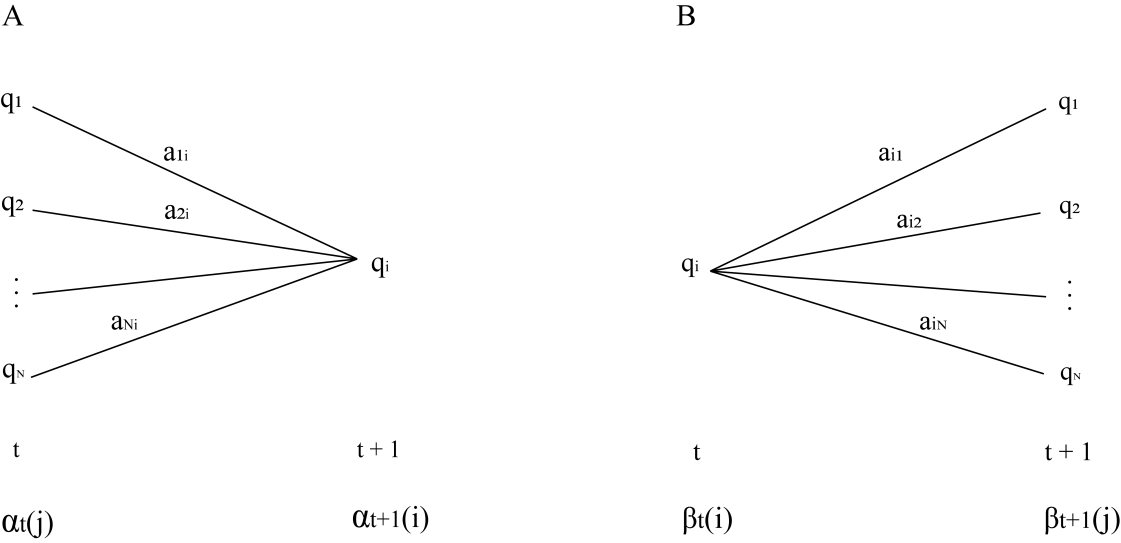


**Figure S2**. The schematic diagram of the recurrence formula for forward and backward probability. (A) The schematic diagram of the forward probability recurrence formula. (B) The schematic diagram of the backward probability recurrence formula.

## *The Forward Algorithm*

Given the HMM parameters, i.e., λ = (*A*, *B*, *π*), if we define the probability that the partial observation sequence from time point 1 to *t* in the HMM as *O* = (*o*_1_, *o*_2_, ... , *o_t_*) and the state in time point *t* is *q_i_* is the forward probability, i.e., *α_t_* (*i*) = *P* (*o*_1_, *o*_2_, ... , *o_t_*, *x_t_* = *q_i_* | λ ). Using the trellis diagram, we can figure out the forward probability and calculate the observation sequence by recursion.

**a.** Initialization.

*α*_1_(*i*) = *π_i_b_i_* (*o*_1_), *i* = 1, 2 , ... , *N*

The first step initializes the forward probability as the joint probability of the state *x*_1_ = *q_i_* and the observation *o*_1_ in the initial time point.

**b.** Recursion, *t* = 1, 2, ... , *T*-1

*α_t_*_+1_(*i*) = [$\sum_{j=1}^{N} \alpha$_t_(*i*)*a_ji_* ] *b_i_* (*o_t_*_+1_) ,*i* = 1, 2, ... , *N*

The second step calculates the forward probability up to the time point *t* + 1 with the partial observation sequence *o*_1_, *o*_2_, ... , *o_t_*, *o_t_*_+1_ and the state *q_i_* in the time point *t* + 1. Here, *α_t_* (*i*) represents the definition of the forward probability, i.e., the probability of the partial observation sequence to time point *t* is *o*_1_, *o*_2_, ... , *o_t_* and the state in the time point *t* is *q_i_*. The product *α_t_*(*i*)*a_ij_* represents the joint probability of the partial observation sequence *o*_1_, *o*_2_, ... , *o*_t_ and the state *q_i_* in *t* and the state *q_j_* in *t* + 1.We sum this product for overall possible states *q_j_* (the length is *N*) in *t*. Multiplying the summed value with the observation probability *b_i_*(*o_t_*_+1_), the resulting product represents the forward probability *α_t_*_+1_(*i*) when the partial observation sequence to *t* + 1, i.e., *o*_1_, *o*_2_, ... , *o_t_*, *o_t_*_+1_ and *q_i_* in *t* + 1. The above is the recursive process of the forward algorithm.

**c.** Termination.

*P* ( *O* | *λ* ) = $\sum_{i=1}^{N} \alpha$*_T_*(*i*)

The third step finally gives the calculating formula for the occurrence probability of the observation sequence *O*. When the time point is *T*, the observation sequence is complete rather than partial. So, the forward probability is the probability of the *α_T_* (*i*) = *P* (*o*_1_, *o*_2_, ... , *o_t_*, *x_T_* = *q_i_* | λ) and *P* ( *O* | λ ) = $\sum_{i=1}^{N} \alpha$*_T_*(*i*).

As a dynamic programming algorithm, the forward algorithm means that a large problem is decomposed into many small problems. The solutions of the small problems are used to obtain the solutions of the large problem. After solving each decomposed small problem, the result is saved for being used directly when needed next time and effectively avoiding the problem of repeated calculations. There are many paths between non-adjacent states but only one path between adjacent states in a trellis diagram. The forward algorithm is based on this feature to recursively calculate the probability of occurrence of the given observation sequence. The forward algorithm calculates the local forward probability in the trellis diagram and uses the path structure to recursively transfer the local forward probability to the global one to obtain *P* ( *O* | λ ). Specifically, *N* values of *α*_1_(*i*) are computed (*i* = 1, 2, ... , *N*) in the time point *t* = 1. Next, recursively following the trellis diagram, *N* values of *α_t_*_+1_(*i*) are computed (*i* = 1, 2, ... , *N*) in each time point *t* = 1, 2, ... , *T*-1 and each *α_t_*_+1_(*i*) is calculated utilizing the *N* values of *α_t_* (*j*) saved from the previous time point. This allows each calculation directly refer to the result of the previous time point and reduce the computational effort and also avoiding the repeated calculations.

## *The Backward Algorithm*

Similar to the forward probability, the backward probability is the probability of the observation subsequence from *t* + 1 to *T* conditioned on the model being in the state *i* in the time point *t*. It is denoted as *β_t_* (*i*) = *P* (*o_t_*_+1_, *o_t_*_+2_, ... , *o_T_* | *x_t_* = *q_i_*, λ).

**a.** Initialization.

*β_T_* (*i*) = 1, *i* = 1, 2, ... , *N*

The observation sequence continues until the time point *T* and terminates. We specify the initial value *β_T_* (*i*) as 1, since this does not affect the calculation.

**b.** Recursion.

When the time point *t* = *T*-1, *T*-2, ... , 1

*β_t_* (*i*) = $\sum_{j=1}^{N} a$_ij_*b_j_* (*o_t_*_+1_) *β_t_*_+1_(*j*)

The second step is to calculate the backward probability by a recursive formula for the backward probability. Unlike the forward probability, the direction of the backward probability recurrence formula is from the back to the front. For calculating the backward probability *β_t_* (*i*), we need to consider the state transition probability of all possible states *q_j_* in the time point *t* + 1, i.e., *a_ij_*, and the observation probability of the observation *o_t_*_+1_ emitted by the state *q_j_*, i.e. *b_j_* (*o_t_*_+1_). The backward probability of the observation sequence after the state *q_j_*, i.e., *β_t_*_+1_(*j*), also should be considered. We can obtain the backward probability *β_t_* (*i*) by multiplying and summing these three terms. By keeping going forward in this way, the state in the time point *t* is known. We can get the states in the time point *t* + 1 by the state transition probability.

**c.** Termination.

*P* ( *O* | λ ) = $\sum_{i=1}^{N} \pi$_i_*b_i_* (*o*_1_) *β*_1_(*i*)

The idea of the third step is basically the same as the second step, except that the state transition probability is replaced by the initial probability.

# Illustrating HMM Through a Three-State Toy Example

It is assumed that there are three boxes and ten balls in each box. The balls are divided into the red and white colors. There are five red balls and five white balls in box one, six red balls and four white balls in box two, and three red balls and seven white balls in box three. We take out the ball according to the following method to generate an observation sequence about color of the balls. Firstly, a box was randomly selected from the three boxes with probability *P*. Secondly, we randomly take out a ball from the selected box with probability *B*, record its color and put it back. Thirdly, it was randomly transferred from the current box to the next box with probability *A*. Next, we randomly take out a ball from this box with probability *B*. Record the color of the ball and put it back. This process is repeated several times to obtain a ball color observation sequence.

We add specific values to facilitate the calculation. It is supposed that the box we first take out is the box one whose probability is 0.4, the probability of the box two is 0.3, and the probability of the box three is 0.3. If the current box is the box one, the probability of it transfers to itself is 0.4, to the box two is 0.3, and to the box three is 0.3; if the current box is the box two, then it transfers to the box one with a probability of 0.5, to the box two with the probability of 0.1, and to the box three with a probability of 0.4; if the current box is the box three, then it transfers to the box one with a probability of 0.2, to the box two with a probability of 0.3 and to itself with a probability of 0.5. Once the transferred box is confirmed, randomly take out a ball from the box, record its color and put it back.

Here, this process was repeated three times to obtain an observation sequence of the color about the balls: *O* = {white, red, white}. In the whole process, the observer can only get the sequence of the color instead of the box which the ball was taken and the sequence of the boxes.


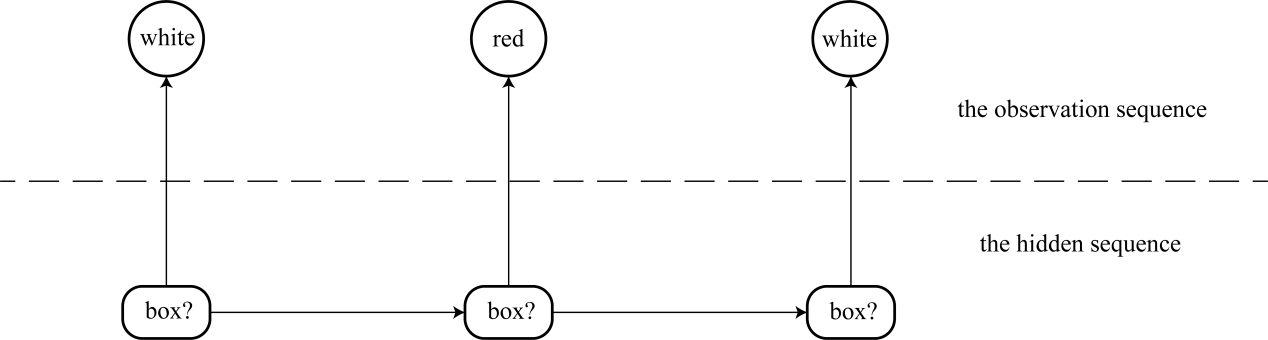


**Figure S3**. Schematic representation of the toy HMM.

Through the analysis of the above problem, we can know there are two random sequences in **Figure S3**. One is the sequence of the boxes as the state sequence and the other is the sequence of the color about the balls as the observation sequence. The state sequence is hidden and can’t be observed, but the observation sequence can be observed. According to the given conditions, we can obtain the state set, observation set, the length of the sequence, and the three elements of the model.

The state set: *Q* = {box1, box2, box3}, *N* = 3

The observation set: *V* = {red, white}, *M* = 2

The state transition probability matrix:

*A* = $\begin{matrix} 0.4 & 0.3 & 0.3 \\ 0.5 & 0.1 & 0.4 \\ 0.2 & 0.3 & 0.5 \end{matrix}$

The observation probability matrix:

*B* = $\begin{matrix} 0.5 & 0.5 \\ 0.6 & 0.4 \\ 0.3 & 0.7 \end{matrix}$

The initial state probability matrix:

*π* = $\begin{matrix} 0.4 \\ 0.3 \\ 0.3 \end{matrix}$


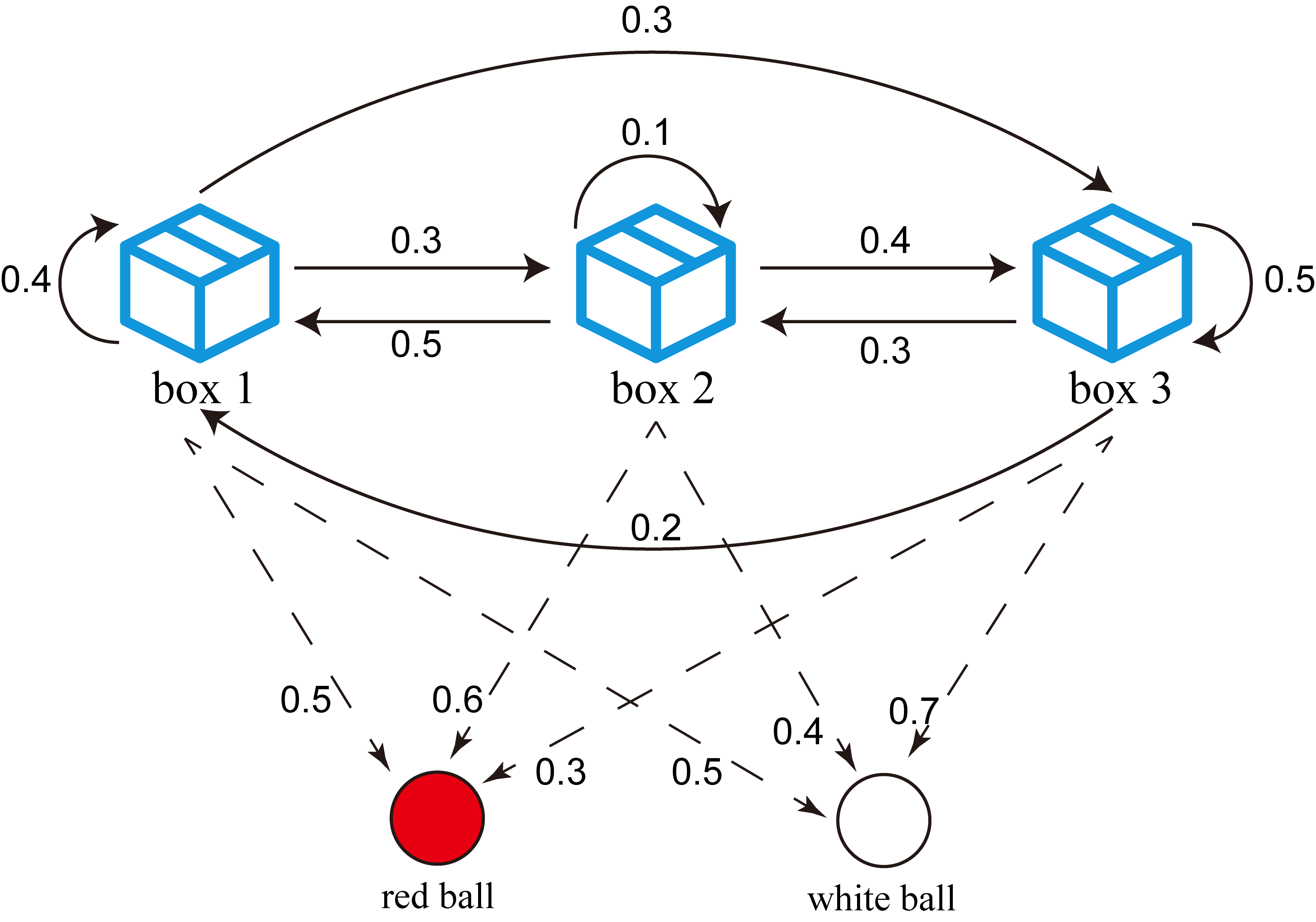


**Figure S4**. Illustration of the HMM with three states.

According to the above parameters, we draw a three-states HMM in **Figure S4**. Three boxes containing different numbers of red and white balls are represented as three states *Q* = {box1, box2, box3}. The states are represented as the box patterns in the illustration. The transitions from one state to another are given as the arrows and represent the transition probability matrix *A*. The transition probabilities are given alongside the arrows. The sum of probabilities arising from it or into it must be 1 for any given state.

After obtaining valuable information, the related problems of HMM can be solved according to the algorithm.

## *Solving the Evaluation Problem with Forward Algorithm*

We can use the forward algorithm to calculate the probability of observing the color sequence *O* = {white, red, white}. This involves iterating through the sequence and applying the algorithm to compute the likelihood of the observed sequence given the model parameters. The specific procedure of the calculation is as follows:

**a.** Initialization.

*ɑ*_1_(1) = *π*_1_*b*_1_(*o*_1_) = 0.4 × 0.5 = 0.2

*ɑ*_1_(2) = *π*_2_*b*_2_(*o*_1_) = 0.3 × 0.4 = 0.12

*ɑ*_1_(3) = *π*_3_*b*_3_(*o*_1_) = 0.3 × 0.7 = 0.21

**b.** Recursion.

When the time point *t* = 2

*ɑ*_2_(1) = [$\sum_{i=1}^{3} ɑ$_1_(i) *a_i_*_1_]*b*_1_(*o*_2_) = [*ɑ*_1_(1)*a*_11_ + *ɑ*_1_(2)*a*_21_ + *ɑ*_1_(3)*a*_31_]*b*_1_(*o*_2_) = 0.091

*ɑ*_2_(2) = [$\sum_{i=1}^{3} ɑ$_1_(i) *a_i_*_2_]*b*_2_(*o*_2_) = [*ɑ*_1_(1)*a*_12_ + *ɑ*_1_(2)*a*_22_ + *ɑ*_1_(3)*a*_32_]*b*_2_(o_2_) = 0.081

*ɑ*_2_(3) = [$\sum_{i=1}^{3} ɑ$_1_(i) *a_i_*_3_]*b*_3_(*o*_2_) = [*ɑ*_1_(1)*a*_13_ + *ɑ*_1_(2)*a*_23_ + *ɑ*_1_(3)*a*_33_]*b*_3_(*o*_2_) = 0.064

When the time point *t* = 3

*ɑ*_3_(1) = [$\sum_{i=1}^{3} ɑ$_2_(i) *a_i_*_1_]*b*_1_(*o*_3_) = [*ɑ*_2_(1)*a*_11_ + *ɑ*_2_(2)*a*_21_ + *ɑ*_2_(3)*a*_31_]*b*_1_(*o*_3_) = 0.045

*ɑ*_3_(2) = [$\sum_{i=1}^{3} ɑ$_2_(i) *a_i_*_2_]*b*_2_(*o*_3_) = [*ɑ*_2_(1)*a*_12_ + *ɑ*_2_(2)*a*_22_ + *ɑ*_2_(3)*a*_32_]*b*_2_(*o*_3_) = 0.022

*ɑ*_3_(3) = [$\sum_{i=1}^{3} ɑ$_2_(i) *a_i_*_3_]*b*_3_(*o*_3_) = [*ɑ*_2_(1)*a*_13_ + *ɑ*_2_(_2_)*a*_23_ + *ɑ*_2_(3)*a*_33_]*b*_3_(*o*_3_) = 0.064

**c.** Termination.

*P*( *O* | λ ) = $\sum_{i=1}^{3} \alpha$_3_(*i*) = *ɑ*_3_(1) + *ɑ*_3_(2) + *ɑ*_3_(3) = 0.131

## *Optimal State Sequence with Viterbi Algorithm*

The observation sequence *O* = {white, red, white}, find the optimal state sequence, i.e., the optimal path *X** = ($x_{1}^{*}$,$x_{2}^{*}$, ... ,$x_{T}^{*}$).

**a.** Initialization.

When the time point *t* = 1,

*δ*_1_(*i*) = $\pi$*_i_b_i_*(*o*_1_) = $\pi$*_i_b_i_*(white), *i* = 1, 2, 3

*δ*_1_(1) = $\pi$_1_*b*_1_(white) = 0.4 × 0.5 = 0.2

*δ*_1_(2) = $\pi$_2_*b*_2_(white) = 0.3 × 0.4 = 0.12

*δ*_1_(3) = $\pi$_3_*b*_3_(white) = 0.3 × 0.7 = 0.21

*Ψ*_1_(*i*) = 0, *i* = 1, 2, 3

**b.** Recursion.

When the time point *t* = 2

*δ*_2_(1) = max_1≤j≤3_[*δ*_1_(j)*a_j_*_1_]*b*_1_(*o*_2_)

= max*_j_*{0.2 × 0.4, 0.12 × 0.5, 0.21 × 0.2} × 0.5

= 0.04

*Ψ*_2_(1) = 1

*δ*_2_(2) = max_1≤j≤3_[*δ*_1_(*j*)*a_j_*_2_]*b*_2_(*o*_2_)

= max*_j_*{0.2 × 0.3, 0.12 × 0.1, 0.21 × 0.3} × 0.6

= 0.0378

*Ψ*_2_(2) = 3

*δ*_2_(3) = max_1≤j≤3_[*δ*_1_(*j*)*a_j_*_3_]*b*_3_(*o*_2_)

= max{0.2 × 0.3, 0.12 × 0.4, 0.21 × 0.5} × 0.3

= 0.0315

*Ψ*_2_(3) = 3

**c.** Termination.

When the time point *t* = 3

*δ*_3_(1) = max_1≤j≤3_[*δ*_2_(*j*)*a_j_*_1_]*b*_1_(*o*_3_)

= max{0.04 × 0.4, 0.0378 × 0.5, 0.0315 × 0.2} × 0.5

= 0.00945

*Ψ*_3_(1) = 2

*δ*_3_(2) = max_1≤j≤3_[*δ*_2_(*j*)*a_j_*_2_]*b*_2_(*o*_3_)

= max{0.04 × 0.5, 0.0378 × 0.1, 0.0315 × 0.3}×0.4

= 0.008

*Ψ*_3_(2) = 1

*δ*_3_(3) = max_1≤j≤3_[*δ*_2_(*j*)*a_j_*_3_]*b*_3_(*o*_3_)

= max{0.04 × 0.3, 0.0378 × 0.4, 0.0315 × 0.5}×0.7

= 0.011025

*Ψ*_3_(3) = 3

The probability of the optimal path: *P** = max_1≤i≤3_*δ*_3_(*i*) = max{0.00945, 0.008, 0.011025} = 0.011025

The end of the optimal path:$x_{3}^{*}$ = argmax_1≤i≤3_*δ*_3_(*i*) = 3

**d.** Backtracking.

Based on the end of the optimal path $x_{3}^{*}$,backtrack to find $x_{2}^{*}$,$x_{1}^{*}$.

When the time point *t* = 2, $x_{2}^{*}$ = *Ψ*_3_($x_{3}^{*}$) = *Ψ*_3_(3) = 3

When the time point *t* =1,$x_{1}^{*}$ = *Ψ*_2_($x_{2}^{*}$) = *Ψ*_2_(3) = 3

The optimal path, i.e., the optimal state sequence *X** = ($x_{1}^{*}$,$x_{2}^{*}$, $x_{3}^{*}$) = (3, 3, 3) = (box3, box3, box3).

# Implementing HMM Algorithms to the Toy Example in R

# Library the hidden Markov model package in R

library(HMM)

# Construct the hidden Markov model

# All possible hidden states

Hidden_states <- c("box1", "box2", "box3")

# All posible observations

Observation_symbols <- c("red", "white")

# The initial probability distribution of the hidden states

Initial_probability <- c(0.4, 0.3, 0.3)

# State transition probability matrix

transition_probability <- matrix(c(0.4, 0.3, 0.3, 0.5, 0.1, 0.4, 0.2, 0.3, 0.5),

nrow = 3, byrow = T)

transition_probability

# [,1] [,2] [,3]

#[1,] 0.4 0.3 0.3

#[2,] 0.5 0.1 0.4

#[3,] 0.2 0.3 0.5

# Observation probability matrix

emission_probability <- matrix(c(0.5, 0.5, 0.6, 0.4, 0.3, 0.7),

nrow = 3, byrow = T)

emission_probability

# [,1] [,2]

#[1,] 0.5 0.5

#[2,] 0.6 0.4

#[3,] 0.3 0.7

# Initialize the hidden Markov model

hmm <- initHMM(States = Hidden_states, Symbols = Observation_symbols,

startProbs = Initial_probability, transProbs = transition_probability,

emissionProbs = emission_probability)

# The observation sequence given in the example

observation_sequence <- c("white", "red", "white")

# Evaluation problem---Forward algorithm

# The probabilities are given on a logarithmic scale (natural logarithm)

logForwardProbabilities <- forward(hmm, observation_sequence)

# The forward-function outputs a matrix containing the forward probabilities

Forward_matrix <- exp(logForwardProbabilities)

Forward_matrix

# index

#states 1 2 3

# box1 0.20 0.0910 0.044840

# box2 0.12 0.0810 0.021828

# box3 0.21 0.0639 0.064155

# Sum the last column of the Forward_matrix

sum(Forward_matrix[,3])

# 0.130823 is the end result of the forward algorithm

# Evaluation problem---Backward algorithm

logBackwardProbabilities = backward(hmm, observation_sequence)

Backward_matrix <- exp(logBackwardProbabilities)

Backward_matrix

# index

#states 1 2 3

# box1 0.2599 0.53 1

# box2 0.2351 0.57 1

# box3 0.2411 0.57 1

# Don't just sum a column of the matrix here, need to do some more calculations

Backward_algorithm_result <- 0.4*emission_probability[1,2]*Backward_matrix[1,1] +

0.3*emission_probability[2,2]*Backward_matrix[2,1] + 0.3*emission_probability[3,2]*Backward_matrix[3,1]

# 0.130823 is the result of the backward algorithm, the same as the forward algorithm

# Decoding problem——-Viterbi algorithm

viterbi(hmm, observation_sequence)

#[1] "box3" "box3" "box3"

# Learning problem—-—Baum-Welch algorithm

# Set initial parameters of the HMM

Init_hmm<-initHMM(States = Hidden_states, Symbols = Observation_symbols, transProbs = transition_probability,

emissionProbs = emission_probability, startProbs = Initial_probability)

print(Init_hmm)

# A vector of observations

a <- sample(c(rep("red", 500), rep("white", 300)))

b <- sample(c(rep("red", 300), rep("white", 500)))

observation <- c(a, b)

# The parameter values will be replaced by those estimated by this function

# Here，only calculate the results after 50 iterations

bw <- baumWelch(Init_hmm, observation, 50)

bw$hmm

#$States

#[1] "box1" "box2" "box3"

#$Symbols

#[1] "red" "white"

#$startProbs

#box1 box2 box3

#0.4 0.3 0.3

#$transProbs

# to

#from box1 box2 box3

# box1 0.5096930 0.3360954 0.1542116

# box2 0.5702258 0.1087915 0.3209826

# box3 0.1036509 0.1857237 0.7106254

#$emissionProbs

# symbols

#states red white

# box1 0.6733013 0.3266987

# box2 0.6863027 0.3136973

# box3 0.2639860 0.7360140

# Vector of differences calculated from consecutive transition and emission matrices in each iteration

bw$difference

#[1] 0.159886453 0.012062673 0.012314951 0.012588421 0.012880874 0.013189785 0.013512084 0.013844081 0.014181382

#[10] 0.014518813 0.014850366 0.015169170 0.015467504 0.015736870 0.015968136 0.016151779 0.016278217 0.016338253

#[19] 0.016323602 0.016227491 0.016045267 0.015774966 0.015417771 0.014978271 0.014464506 0.013887726 0.013261933

#[28] 0.012603225 0.011929069 0.011257633 0.010607320 0.009996657 0.009444648 0.008971416 0.008598193 0.008344084

#[37] 0.008217354 0.008206747 0.008284848 0.008421353 0.008592441 0.008782380 0.008981657 0.009184821 0.009388933

#[46] 0.009592591 0.009795325 0.009997243 0.010198795 0.010400644

# Supplementary introduction of HMM related tools

## *Transmembrane protein prediction*

As a statistic model, the HMM is supported by perfect mathematical theory. Through learning from the training data, the distribution information of each amino acid in TM protein can be obtained.^[1](#_ENREF_1)^ The basic principle of modeling TM proteins with the HMM is defining a set of states that correspond to different regions of the TM protein. The simplest model may consist of three states: an inner loop, a TM region, and an outer loop. Each state has a probability distribution of 20 amino acids, indicating the variability of amino acids in the modeled region. The states are connected in a biologically plausible manner, and a learning algorithm can learn the transition probabilities and amino acid emission probabilities between states in the HMM.

*HMMTOP*

HMMTOP (https://hmmtop.pbrg.hu/) is an automatic server based on the HMM for predicting TM helices and the topology of proteins. Tusnády GE and Simon I devised the method to design the server in 1998. It was based on the idea that the maximum difference in the distribution of amino acids from diverse structural parts of the membrane protein determines topology. Therefore, the HMM in HMMTOP is divided into five structural states: inside loop state, inner helix tail state, membrane helix state, outer helix tail state, and outside loop state. The prediction process of the model is divided into the following stages: first, the initial estimates of the parameters of the HMM are set, including the initial state, observation sign, and state transfer probability; next, these parameters are optimized by Baum-Welch algorithm; and finally, the best state sequence is found by the Viterbi algorithm for a given observation sequence.^[2](#_ENREF_2)^ In 2001, HMMTOP released version 2.0, which had two significant improvements compared to the previous version. The first one is that the program code has been redesigned to apply multiple HMM architectures flexibly. In addition, the speed of topology prediction of TM protein has been significantly improved. The second improvement is to use the preliminary experimental information as the limiting condition. The user can add the specific positioning information of the five states in the sequence during the prediction process. It dramatically improves prediction accuracy.^[3](#_ENREF_3)^ HMMTOP is the first server to consider experimental constraints in the prediction.^[4](#_ENREF_4)^ The users only need to paste the protein sequence as input files into the specified text area in three formats: plain text, FASTA, and NBRF/PIR.

Meanwhile, the server can handle the submitted sequences as single or homologous sequences and run reliably or quickly. If the localization of some parts in the protein sequence queried by the user is known, the server can add it as a constraint to the prediction process. The output can be generated as an HTML or simple text file. The file mainly contains the length of the submitted TM protein sequence, the position of the N-terminus relative to the membrane, the number of TM helices and their specific position interval in the sequence, and the best path of the whole sequence predicted by the model.

*HMM-TM*

Adding rude conditions such as prior topological information to the HMM results in the loss of probabilistic interpretation. In the past, little effort has been made to describe this case. Although some studies have considered this case, no corresponding algorithm details have been proposed.^[5](#_ENREF_5);[6](#_ENREF_6)^ Pantelis G Bagos et al. first introduced modifications to the standard algorithms and published the details. These modifications include the forward and backward algorithms for *the Evaluation problem* and the Viterbi, 1-best,^[7](#_ENREF_7)^ posterior, and Posterior-Viterbi^[8](#_ENREF_8)^ for the Decoding problem. It is noted that it does not confer bias to the prediction method, although the algorithm has been modified. The HMM architecture in HMM-TM is similar to that in HMMTOP.^[2](#_ENREF_2)^ It consists of three sub-models denoted by the labels: Cytoplasmic loop, TM helix, and Extracellular loop. The model is cyclic and consists of 114 states, which accords with the actual biological structure of TM proteins. HMM-TM is freely available for academic users, and any of the four algorithms above can be selected. The TM segments of alpha-helical membrane proteins can be predicted after pasting the protein sequence in FASTA format into the specified text area. Users can submit the specific start and end positions of different functional regions (in, TM, out) of the predicted TM protein sequence as prior knowledge. Output uses three different labels (in, tm, out) and the same number of distinct colors to define the triple-state, in which a residue can be: [in] for periplasmic space, [tm] for TM strand, and [out] for extracellular space. Each output shows the specific start and end locations of three different spaces of the TM protein and comes with a graphical plot combining all individual predictions. The plot also supports a custom mode that allows users to adjust the label lines' length, width, and visibility.

*HMMpTM*

In addition to adding prior information in the prediction process, combined prediction is another common method to improve the prediction accuracy of the topological structure of TM proteins. Prediction algorithms can use this information to predict TM proteins’ orientation more effectively. The HMM used in HMMpTM is quite similar to the one proposed by HMM-TM. But HMMpTM has two additional sub-models: the Phosphorylation Site sub-model and the Glycosylation Site sub-model. Due to their being compartment-specific, the two sub-models are connected with the Cytoplasmic Loop and the Extracellular Loop sub-models, respectively.^[9](#_ENREF_9)^ HMMpTM not only improves the accuracy of the prediction of TM proteins but also provides a reliable prediction of glycosylation and phosphorylation sites to a certain extent. Of note, the input protein sequence must be in FASTA format. The users can select a certain score threshold for the prediction results and click the submit button to execute the prediction. The final results are mainly divided into TM protein topology prediction and glycosylation and phosphorylation sites prediction. The format of topology prediction results is similar to those in HMM-TM.^[10](#_ENREF_10)^ HMMpTM provides the names of protein sequences submitted by users in the prediction results and different colored labels (i, o, m) to mark the topology of the predicted proteins. In addition, each prediction topology includes a reliability score. HMMpTM also provides a posteriori-label probability plot generated by Gnuplot ([http://www.gnuplot.info/](http://www.gnuplot.info/)), which can be downloaded as an SVG file. In the phosphorylation and glycosylation prediction results, modified residues are represented by different lowercase letters. HMMpTM provides the exact location along the protein sequence of amino acid residues predicted to be modified  and corresponding kinases for phosphorylation sites. Each site is provided with a score calculated using the posterior probability. All of the predictions can be downloaded in a text file.

*Phobius*

Phobius first combined the prediction of TM helices and SPs into a unified HMM. The HMM in Phobius consists of the TM helix sub-model, the SP sub-model, the cytoplasmic sub-model, and the non-cytoplasmic sub-model. The TM topology and SP model combination allow Phobius to improve the distinction between TM and SP. The presence of SP reveals the orientation of TM concerning the membrane, thus simplifying the TM topology prediction problem.^[11](#_ENREF_11);[12](#_ENREF_12)^ As homologous sequences likely share TM topology and SPs, Phobius further derived a new method-ployphobius. It significantly improves the prediction performance due to the incorporation of information from the homologs.^[13](#_ENREF_13)^ Phobius takes proteins in FASTA format. The output includes a list of the location of the predicted TM helices, the predicted location of the intervening loop regions and SP, and a plot showing the posterior probability of cytoplasmic, non-cytoplasmic, TM helices, and SPs. Phobius allows users to add prior knowledge and homolog information to the prediction.

## *Gene finding*

Currently, there are many gene identification methods, of which statistical^[14](#_ENREF_14)^ and homologous^[15](#_ENREF_15)^ are the two main types. The statistical method is also known as the ab initio gene search algorithm, which uses the composition characteristics of genome sequence to grasp the internal rules and characteristics of genes for statistical analysis to identify genes.^[16](#_ENREF_16)^ These properties include codon bias, periodicity, and dependence among the bases of the coding region. Based on local alignment methods such as the BLAST family of programs, the homology method makes gene predictions based on the results of comparison with annotated gene sequences.^[17](#_ENREF_17)^ HMM is mainly used in statistical methods, and many prediction tools have been developed.^[18](#_ENREF_18);[19](#_ENREF_19)^

The HMM can predict discrete sequences commendably. In earlier studies, Markov chains were used to analyze coding and non-coding regions of genes.^[20](#_ENREF_20);[21](#_ENREF_21)^ For example, GeneMark identifies genes in E.coli by independently modeling the coding and non-coding regions.^[22](#_ENREF_22)^ Generally, a gene includes promoters, start codons, exons, introns, stop codons, and untranslated regions at both ends. Suppose a given genomic DNA sequence is taken for an observation sequence. In that case, gene prediction can be regarded as a hidden Markov process and the gene structure as a hidden state which cannot be observed directly. Therefore, gene identification becomes a problem that predicts the optimal state path of a gene structure based on a given genomic DNA sequence. Firstly, the key to solving this problem is constructing the HMM for gene recognition. Different HMMs should be designed because of natural species’ different regularity of DNA sequences. For example, A Krogh et al. used HMMGene for gene prediction of genomic DNA sequences of E.coli in 1994.^[23](#_ENREF_23)^ John Henderson et al. used VEIL (Viterbi Exon-Intron Locator) for eukaryotic gene prediction in 1997.^[24](#_ENREF_24)^ Secondly, through the statistical analysis of many annotated genomic DNA sequences, the state transition patterns between genes’ coding and non-coding regions and the distribution patterns of their internal bases or codons are derived. Thirdly, the corresponding state transition matrices and observation probability matrices are made. The models are fitted by the maximum likelihood method or the Baum-Welch algorithm. As regulation of gene expression in eukaryotes is a very complex process, it is necessary to individually model specific regions of genes and integrate these sub-hidden Markov models into a large HMM. Finally, the Viterbi algorithm is required for the prediction process to integrate segmented genes in intergenic regions into a coherent parse.^[25](#_ENREF_25)^

*GENSCAN*

It differs from most existing prediction programs in three significant ways. First, GENSCAN uses an explicit double-stranded genome sequence model to make simultaneous predictions about potential genes on both positive and negative strands of DNA; Second, GENSCAN can handle the situation where the input sequence contains a partial gene, a complete gene, and multiple complete or partial genes while most complete gene-discovery programs presuppose that the input genomic DNA sequence contains at least one complete gene; Third, the program describes a new model of donor and receptor splicing signals. The new approach, called maximum dependence decomposition, is also introduced to model functional signals in DNA (or protein) sequences and allows for the dependence between signal locations in a relatively natural and statistically reasonable manner.^[26](#_ENREF_26)^

*AUGUSTUS*

The input can be one or more pasted DNA sequences or an uploaded file in FASTA format. The result consists of the protein-coding parts of the genes and the amino acid sequences of the predicted genes. The program outputs its result in both graphics and text format. AUGUSTUS may predict no gene, one or more genes. Besides, AUGUSTUS allows the user to impose constraints to predict an exon, a splice site, a translation start, or a translation end site at a certain position in the sequence.

*GeneMark*

The initial idea is to construct specific sub-models for DNA coding and non-coding region, respectively, and combine them with the Bayesian decision function to generalize the high-order Markov chain model. Meanwhile, it also simultaneously models both the positive and negative strands of DNA to analyze them, which was considered an innovation then. The GeneMark family includes two main programs, GeneMark^[27](#_ENREF_27)^ and GeneMark.hmm.^[28](#_ENREF_28)^ They model the coding region using a non-flush Markov chain model and the non-coding region with a flush Markov chain model. It is a local method that uses a Bayesian form to calculate the posterior probability of the presence of genetic code in short DNA sequence fragments. GeneMark.hmm uses the HMM framework and a modified Viterbi algorithm for gene prediction. Compared with GeneMark, Genemark.hmm is more comprehensive and can predict genes in prokaryotes, eukaryotes, and metagenomes. In addition to the two main gene prediction programs, the GeneMark family also includes some genetic prediction aids and information integration and provides a link to download the prediction tools to provide convenience for those who need scientific research.

*HMMGene*

Users can either submit a local file with sequences in FASTA format or paste the sequence into the window. The output is a prediction of partial or complete genes in the sequences of [GFF](https://www.sanger.ac.uk/resources/software/gff/spec.html) format, a sequence annotation format developed with gene finding in mind. HMMGene enhances the algorithm of the HMM by using conditional maximum likelihood to train the model^[7](#_ENREF_7)^ to improve the prediction accuracy of the model as well as the sensitivity and specificity of exons. The N-Best algorithm, an alternative to the commonly used Viterbi algorithm, encodes the model. The N-best algorithm can find multiple state paths, while the Viterbi algorithm can only find the most probable one. Using conditional maximum likelihood estimation models is necessary because the same prediction result has many possible state paths.

## *Sequence alignment*

As a probabilistic model, HMM is suitable for modeling linear data such as biological sequences and performs well in sequence comparison. According to the sequence alignment process, the results of residue alignment can be divided into match, insertion, and deletion, which can be regarded as the hidden states in the HMM. The probability of residue occurrence is also different in the diverse positions of the sequence. As a general extension of HMM for sequence alignment, the profile HMM is commonly used to perform homology searches of protein families.^[29](#_ENREF_29);[30](#_ENREF_30)^ The profile here refers to the statistical expression profile of features, which provides a description common to all protein family members, i.e., the probability of distribution of residues in each column and transfer between different states.^[31](#_ENREF_31)^ Profile HMM is the most classical model in multiple sequence alignment. Many sequence alignment tools based on this model have been developed.

## *CpG island prediction*

*DMRMark*

The non-homogeneous Hidden Markov model (NHMM) can model spatial correlations between CGIs and call DMRs using the Viterbi algorithm. The novel constrained Gaussian Mixture Mode models the M-value pairs of each locus. The two significant advantages of DMRMark are the automatic DMR calling and the ability to detect DMRs without replicates. However, despite the abovementioned advantages, it only handles the two-group comparison. By converting methylation ratios to β-values, Whole Genome Bisulfite Sequencing (WGBS) data can also be applied to DMRMark. In addition, parallelization can significantly reduce the calculation time of DMRMark.^[32](#_ENREF_32)^

*BSDMR*

It considers the effect of genomic distance on the correlation of methylation levels of adjacent CpGs and the relationship of methylation signals between normal and disease samples. The NHMM in the BSDMR is a four-state HMM, including hypermethylation and hypomethylation, both highly methylated and both highly unmethylated. BSDMR is also applicable to Reduced Representation Bisulfite Sequencing (RRBS) data. As a Bayesian method, BSDMR has strict computing power and storage space requirements. Therefore, when the amount of data to be processed is large, dividing the data set into subsets and processing them in the sequence is recommended.^[33](#_ENREF_33)^

## *Copy number variation detection*

Relying on Hidden Markov Models, researchers have invented many methods for detecting CNVs. Most of them are based on this idea. Suppose the exonic region of a sample genome has a copy number gain or loss relative to the reference genome. In that case, the number of reads in sequencing data will also change accordingly. That is, according to the ratio of the number of observed reads to the number of expected reads, we can derive the change in copy number. Therefore, the most basic HMM for detecting CNVs has three hidden states: normal, loss, and gain. We can use HMM to identify abnormal reads’ states to detect copy number variation.

#

# References

1. Tsirigos KD, Govindarajan S, Bassot C, et al. Topology of membrane proteins-predictions, limitations and variations. *Curr Opin Struct Biol.* 2018;50:9-17.

2. Tusnády GE, Simon I. Principles governing amino acid composition of integral membrane proteins: application to topology prediction. *J Mol Biol.* 1998;283(2):489-506.

3. Tusnády GE, Simon I. The HMMTOP transmembrane topology prediction server. *Bioinformatics.* 2001;17(9):849-850.

4. Tusnády GE, Kalmár L, Simon I. TOPDB: topology data bank of transmembrane proteins. *Nucleic Acids Res.* 2008;36(Database issue):D234-239.

5. Melén K, Krogh A, von Heijne G. Reliability measures for membrane protein topology prediction algorithms. *J Mol Biol.* 2003;327(3):735-744.

6. Bernsel A, Von Heijne G. Improved membrane protein topology prediction by domain assignments. *Protein Sci.* 2005;14(7):1723-1728.

7. Krogh A. Two methods for improving performance of an HMM and their application for gene finding. *Proc Int Conf Intell Syst Mol Biol.* 1997;5:179-186.

8. Fariselli P, Martelli PL, Casadio R. A new decoding algorithm for hidden Markov models improves the prediction of the topology of all-beta membrane proteins. *BMC Bioinformatics.* 2005;6 Suppl 4(Suppl 4):S12.

9. Tsaousis GN, Theodoropoulou MC, Hamodrakas SJ, Bagos PG. Predicting Alpha Helical Transmembrane Proteins Using HMMs. *Methods Mol Biol.* 2017;1552:63-82.

10. Bagos PG, Liakopoulos TD, Hamodrakas SJ. Algorithms for incorporating prior topological information in HMMs: application to transmembrane proteins. *BMC Bioinformatics.* 2006;7:189.

11. Käll L, Krogh A, Sonnhammer EL. A combined transmembrane topology and signal peptide prediction method. *J Mol Biol.* 2004;338(5):1027-1036.

12. Käll L, Krogh A, Sonnhammer EL. Advantages of combined transmembrane topology and signal peptide prediction--the Phobius web server. *Nucleic Acids Res.* 2007;35(Web Server issue):W429-432.

13. Käll L, Krogh A, Sonnhammer EL. An HMM posterior decoder for sequence feature prediction that includes homology information. *Bioinformatics.* 2005;21 Suppl 1:i251-257.

14. Claverie JM. Computational methods for the identification of genes in vertebrate genomic sequences. *Hum Mol Genet.* 1997;6(10):1735-1744.

15. Gish W, States DJ. Identification of protein coding regions by database similarity search. *Nat Genet.* 1993;3(3):266-272.

16. Burge CB, Karlin S. Finding the genes in genomic DNA. *Curr Opin Struct Biol.* 1998;8(3):346-354.

17. Altschul SF, Madden TL, Schäffer AA, et al. Gapped BLAST and PSI-BLAST: a new generation of protein database search programs. *Nucleic Acids Res.* 1997;25(17):3389-3402.

18. Burge C, Karlin S. Prediction of complete gene structures in human genomic DNA. *J Mol Biol.* 1997;268(1):78-94.

19. Krogh A. Using database matches with for HMMGene for automated gene detection in Drosophila. *Genome Res.* 2000;10(4):523-528.

20. Churchill GA. Stochastic models for heterogeneous DNA sequences. *Bull Math Biol.* 1989;51(1):79-94.

21. Tavaré S, Song B. Codon preference and primary sequence structure in protein-coding regions. *Bull Math Biol.* 1989;51(1):95-115.

22. Kleffe J, Hermann K, Borodovsky M. Statistical analysis of GeneMark performance by cross-validation. *Comput Chem.* 1996;20(1):123-133.

23. Krogh A, Mian IS, Haussler D. A hidden Markov model that finds genes in E. coli DNA. *Nucleic Acids Res.* 1994;22(22):4768-4778.

24. Henderson J, Salzberg S, Fasman KH. Finding genes in DNA with a Hidden Markov Model. *J Comput Biol.* 1997;4(2):127-141.

25. Snyder EE, Stormo GD. Identification of coding regions in genomic DNA sequences: an application of dynamic programming and neural networks. *Nucleic Acids Res.* 1993;21(3):607-613.

26. Burge CBJNCB. Chapter 8 Modeling dependencies in pre-mRNA splicing signals. 1998;32:129-164.

27. Borodovsky M, Mcininch JJC, Chemistry. GENMARK: Parallel gene recognition for both DNA strands. 1993;17(2):123-133.

28. Lukashin AV, Borodovsky M. GeneMark.hmm: new solutions for gene finding. *Nucleic Acids Res.* 1998;26(4):1107-1115.

29. Eddy SR. Profile hidden Markov models. *Bioinformatics.* 1998;14(9):755-763.

30. Pattabiraman S, Warnow T. Profile Hidden Markov Models Are Not Identifiable. *IEEE/ACM Trans Comput Biol Bioinform.* 2021;18(1):162-172.

31. Krogh A, Brown M, Mian IS, Sjölander K, Haussler D. Hidden Markov models in computational biology. Applications to protein modeling. *J Mol Biol.* 1994;235(5):1501-1531.

32. Shen L, Zhu J, Robert Li SY, Fan X. Detect differentially methylated regions using non-homogeneous hidden Markov model for methylation array data. *Bioinformatics.* 2017;33(23):3701-3708.

33. Chen Y, Kwok CK, Jiang H, Fan X. Detect differentially methylated regions using non-homogeneous hidden Markov model for bisulfite sequencing data. *Methods.* 2021;189:34-43.
